# Data Augmentation for Enhanced Fish Detection in Lake Environments: Affine Transformations, Neural Filters, SinGAN

**DOI:** 10.3390/ani15101466

**Published:** 2025-05-19

**Authors:** Kidai Watanabe, Thao Nguyen-Nhu, Saya Takano, Daisuke Mori, Yasufumi Fujimoto

**Affiliations:** 1 Laboratory of Advanced Technology Applications, Miyagi University, 2-2-1 Hatatate, Taihaku-ku, Sendai City 982-0215, Miyagi, Japan; 2 Graduate School of Food, Agricultural and Environmental Sciences, Miyagi University, 2-2-1 Hatatate, Taihaku-ku, Sendai City 982-0215, Miyagi, Japan; 3 The Miyagi Prefectural Izunuma-Uchinuma Environmental Foundation, 17-2 Shikimi, Wakayanagi, Kurihara 989-5504, Miyagi, Japan; fjimo@hotmail.com

**Keywords:** AI-generated image, anomaly detection, electric shocker boat, habitat, PaDiM

## Abstract

Understanding fish habitats is essential for fisheries management, habitat restoration, and species protection. Automated fish detection is a key tool in these applications, which enables real-time monitoring and quantitative analysis. Recent advancements in high-resolution cameras and machine learning technologies have facilitated image analysis automation, promoting remote fish tracking. However, many of these detection methods require large volumes of annotated data, which involve considerable effort and time. Additionally, their practical implementation remains challenging in environments with limited data. Hence, this study proposes an anomaly-based fish detection approach by integrating Patch Distribution Modeling with data augmentation techniques, including Affine Transformations, Neural Filters, and SinGAN. Field experiments were conducted in Lake Izunuma-Uchinuma, Japan, using an electrofishing boat to acquire data. Evaluation metrics, such as AUROC and F1-score, assessed detection performance. The results indicate that, compared to the original dataset (AUROC: 0.836, F1-score: 0.483), Neural Filters (AUROC: 0.940, F1-score: 0.879) and Affine Transformations (AUROC: 0.942, F1-score: 0.766) improve anomaly detection. However, SinGAN exhibited no measurable enhancement, indicating the necessity for further optimization. This shows the potential of the proposed approach to enhance automated fish detection in limited-data environments, supporting aquatic ecosystem sustainability.

## Simple Summary

Understanding where fish live is key to protecting aquatic environments. This study introduces an automated method that enhances images, even with limited data, to detect fish accurately. Combining a specialized detection model with techniques, such as geometric rotations, photo filters, and AI-generated images, this approach promises faster and more reliable fish monitoring, supporting conservation and sustainable fisheries.

## 1. Introduction

The study of fish habitats has long been a key ichthyological topic. Understanding fish habitats contributes to varied scientific fields, such as ecological research, fisheries management, habitat restoration, conservation efforts, and the protection of endangered species [1]. Research on fish habitats has utilized field observation [2,3] and fish capture [4,5,6] for many years. These methods have provided insights into ecologically significant aspects, including micro- and meso-scale habitat assessments, seasonal distributional changes, and ontogenetic habitat shifts.

Recent advancements in imaging devices and IT technologies, such as underwater cameras [7,8,9] and acoustic imaging sonar [10,11], and environmental DNA analysis [12], have shown great research potential. These methods simplify data collection and enable large-scale monitoring as they offer high detection sensitivity. By providing substantial amount of information efficiently, they demonstrate utility in fish habitat studies.

Building on these innovations, the efficient collection of large amount of high-precision data has paved the way for the development of automated biological detection technologies [13,14]. Particularly, deep learning technology has progressed rapidly, driving significant advancements in object detection by enabling precise identification and object localization across multiple classes [15,16]. It has included underwater fish detection, delivering significant results in marine environments [17] and aquaculture settings [18].

Among deep learning algorithms, YOLO (You Only Look Once) has gained widespread attention as an object detection method that combines efficiency and high accuracy [19]. This algorithm has been successfully applied to underwater environments, detecting diverse fish species in Norwegian fjords and oceans [20]. However, YOLO requires a considerable amount of labeled data, posing significant challenges regarding cost and labor-intensive annotation efforts. To address these limitations, Patch Distribution Modeling (PaDiM), an anomaly detection technique, was designed to maintain high detection accuracy despite scarce labeled data.

Berg et al. [21] introduced an optimized version of PaDiM, which efficiently detected marine organisms in a weakly supervised environment. Despite utilizing a relatively small dataset, it outperformed existing techniques regarding F1 score and recall. Nevertheless, underwater-specific challenges, including lighting variations, foreground camouflage, and dynamic background interference, continue to impede model performance, indicating the necessity for further advancements in detection methodologies.

This study proposes an approach that combines data augmentation techniques with a PaDiM-based automatic fish identification system to improve detection. The proposed system facilitates the efficient monitoring of aquatic organisms to support fisheries’ resource management and ecosystem conservation. This is expected to support the long-term sustainability of aquatic ecosystems.

Additionally, this study prioritizes the development of an anomaly detection model capable of accommodating various factors, including seasonal and temporal variations in lighting conditions, aquatic plant phenology, and dynamic changes in water surface patterns, even under limited-data conditions. To address these challenges, data augmentation techniques were employed using Neural Filters (NF), Affine Transformations (AT), and SinGAN, resulting in an expanded dataset. These augmented datasets were subsequently used to construct a PaDiM-based model, followed by a comprehensive evaluation of its detection accuracy. In conclusion, this study develops a system capable of accurately acquiring information on fish species, population sizes, and spatial distribution while presenting innovative practical approaches for the implementation of electrofishing boat surveys.

## 2. Methods

### 2.1. Study Site

Field surveys were conducted at Lake Izunuma-Uchinuma (38°43’N, 141°07’E), located in northeastern Japan. The lake has a surface area of 387 hectares and a shoreline of approximately 20 km in length. Parts of the shoreline are covered by plant communities dominated by the common reed (*Phragmites australis* (Cav.) Trin. ex Steud.) [22,23]. The lake is shallow, with an average depth of 0.77 m and a maximum depth of 1.6 m, and its surface is extensively covered by floating-leaved macrophytes, including lotus (*Nelumbo nucifera* Gaertner, 1806) and water chestnut (*Trapa* spp.) [24].

Lake Izunuma-Uchinuma is an important freshwater fish habitat in Japan and exhibits a fish fauna that reflects the formation history of eastern Japan [25]. Moreover, it is the primary habitat for small-scale bitterling (*Acheilognathus typus* (Bleeker, 1863)), a rare native species of eastern Japan [26]. Consequently, vigorous conservation activities for preserving the native fish assemblage, including extensive field surveys and invasive largemouth bass (*Micropterus nigricans* (Cuvier, 1828)) removal efforts, have been conducted [27,28]. Due to its high capture efficiency, an electrofishing boat has been employed for the removal of largemouth bass [28,29]. The electrofishing boat, which is 3.3 m in length, is equipped with a generator and transformer (2.5GPP; Smith Route). It delivers approximately 300 V of electricity while in motion, stunning fish within a diameter of about 4 m, causing them to float to the surface.

### 2.2. Dataset

**Figure 1:**
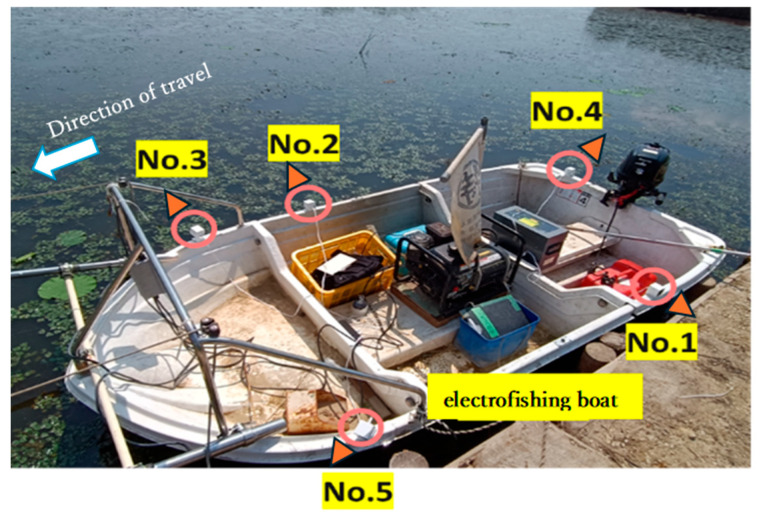
Camera placement on the electrofishing boat. The numbers indicate the corresponding camera numbers. The cameras were either perpendicular to or facing backward relative to the boat’s travel direction. This arrangement allowed comprehensive observation of the surroundings, including the area behind the boat after its passage. The boat’s travel direction was oriented to the left of the image.

This study recorded videos of the water surface of Lake Izunuma-Uchinuma on the morning of 7 June 2023, using five ATOM Cam 2 units (manufactured by ATOM tech Inc., Clarkston, MI, USA). The device was deployed in a water depth of approximately 50 cm, located 20–50 m offshore. Each camera had a resolution of 1920 × 1080 pixels and a frame rate of 20 fps. The cameras were mounted on the edge of an electrofishing boat for recording (Figure 1).

The footage from ATOM Cam 2 No. 5 was selected as it captured the highest number of fish appearances. The video was converted into individual frames, and a random sample of images was extracted. The extracted images were cropped to remove elements, such as electrodes, date stamps, and the sky, ensuring that only the water surface remained. The images containing fish were classified as “anomalous images,” while those without fish were classified as “normal images.” The dataset was designated as the original dataset (hereafter referred to as “Org”) and consisted of both normal and anomalous images. Furthermore, a validation dataset was constructed using the same method as for Org, ensuring that there was no overlap between the datasets (Table 1).

Following the procedure for the Org dataset, a test dataset of 100 fish-containing images was constructed from the video from ATOM Cam 2 No. 1, with each image cropped to retain only the water surface (Table 1).

### 2.3. PaDiM

This study adopted PaDiM [30] as an anomaly detection model capable of detecting freshwater fish in a limited data environment. Detecting fish on the water surface is challenging because their color and morphology are highly diverse and fish appear infrequently, resulting in severely limited training samples. Preliminary experiments showed that supervised object detection methods such as YOLO yielded low recall and precision under these conditions and failed to deliver sufficient performance. In contrast, one-class anomaly detection approaches like PaDiM learn the distribution of “normal” water surface images and detect fish as deviations, achieving high accuracy even with scarce data. This model learns the local feature distribution in the feature space of normal data on a patch-by-patch basis and determines whether unknown data deviates from that distribution. PaDiM evaluates the feature space distribution using the Mahalanobis distance to generate an anomaly score. This enables efficient detection, low computational cost, and flexibility, as there is no need to predefine the location or type of anomalies.

For training PaDiM in this study, the following hyperparameters were uniformly set: wideresnet50_2 was used as the backbone, the threshold was set to adaptive, the batch size was 1, and the number of epochs for both initial training and additional training was 1 each.

### 2.4. Data Augmentation

Data augmentation is widely used to increase training data diversity, suppress overfitting, and improve machine learning models’ accuracy. In image recognition, techniques such as random cropping and horizontal flipping enhance dataset variation, leading to improved model performance and robustness.

This study applied data augmentation using AT, NF, and SinGAN. Each method enhances data diversity in different ways and has been effective in the development of anomaly detection techniques.

#### 2.4.1. Affine Transformation

AT is a geometric transformation technique that can uniformly apply operations, such as rotation, scaling, translation, and shearing. It has been widely used in machine learning fields, including object detection and anomaly detection [31]. In this study, data augmentation using AT was conducted to enhance the generalization performance of the aquatic organism detection model.

The Org dataset was used, and random rotations between 1° and 359°, vertical flipping, horizontal flipping, and both vertical and horizontal flipping were applied. During data augmentation using flipping operations, the same number of normal and anomalous images was obtained as in Org. However, when applying rotation, portions that extended beyond the original frame size were trimmed, potentially removing fish present in those areas. Therefore, images in which fish became unrecognizable due to this issue were excluded from the anomalous image set. As a result, after data augmentation with rotation, the dataset contained 3902 normal images and 36 anomalous images.

#### 2.4.2. Neural Filter

As a data augmentation technique for the anomaly detection model, this study utilized the landscape NF incorporated in the image editing software Adobe Photoshop, provided by Adobe Inc. (San Jose, CA, USA). This tool is designed based on deep learning technology and allows computers to intuitively adjust image attributes, such as “time of day” and “season pattern.” The specific settings for data augmentation in this study are as follows.

For “time of day,” three conditions were applied—daytime, night-time, and evening. Each condition was further adjusted using three intensity levels—30, 60, and 90. For the “season pattern,” four conditions were applied—spring, summer, autumn, and winter—with three intensity levels—30, 60, and 90—for each season. Hence, three intensity variations were applied to each time of day and season, generating 21 new images, with three images each for daytime, night-time, evening, spring, summer, autumn, and winter.

A setting was applied that combined the “time of day” with the “seasonal pattern”. For example, combinations included “daytime & spring” and “nighttime & winter,” and three intensity levels (30, 60, 90) were applied to each combination. This resulted in 108 new images from a single original image.

These augmentation settings were applied to the processing dataset (hereafter referred to as NFbase) using the landscape NF, resulting in 10,578 images. The procedure for constructing the NFbase dataset is as follows. Anomalous images were obtained by converting videos captured by ATOM Cam2 No. 5 on the same day as the training data into images, selecting 20 frames containing fish. Normal images were extracted from videos captured by the same camera, selecting 62 frames. Among these, one frame containing a fish was reclassified as an anomalous image, leading to 21 anomalous images and 61 normal images in the NFbase dataset. Details of the NFbase dataset are presented in Table 1.

After processing with the NF, the images underwent the same trimming process as the training data, removing any images in which fish were not visible. The resolution of all processed and trimmed images was standardized to 1390 × 550 pixels. The final dataset consisted of 7869 normal images and 2064 anomalous images.

#### 2.4.3. SinGAN

Data augmentation using SinGAN was employed to generate multiple similar images from a single image, thereby enhancing the training data. SinGAN is an unconditional generative model that can generate high-quality, diverse images based on a single natural image [32]. This model does not require external datasets and can generate images with different structures while preserving visual features by learning patch statistics from the input image. In this study, this characteristic was utilized to improve the training data diversity.

The experiment for data augmentation using SinGAN in this study was conducted using the Google Colaboratory environment. The runtime was configured with the Ubuntu 22.04.3 LTS (Jammy Jellyfish) operating system, and Python 3.10.12 was used. The GPU allocated was an NVIDIA Tesla T4, with CUDA version 12.2 and Torch version 2.5.0+cu124.

The process of utilizing SinGAN for data augmentation involved the following steps. The SinGAN code was cloned from GitHub, and the necessary files were retrieved. The images were loaded using PIL, resized, and saved for processing. The PyTorch framework, along with the SinGAN model, was used to generate new images based on the given input. Additionally, when required, TensorBoardX was employed to visualize the progress of the generation process.

To prepare input images for SinGAN, the water surface portion of the normal images in the NFbase was cropped, and the green color component was analyzed based on the HSV format. The hue, saturation, and value ranges were set as [35,40] to [85,255], respectively. Based on the pixel percentage within this range, 5 images with green color components of 24.6%, 14.9%, 11.1%, 5.3%, and 0.36% were selected.

The selected 5 images were used as input data for SinGAN, with independent learning conducted for each. During learning, the input images (1920 × 1080 pixels) were resized to 500 × 500 pixels, and training was performed for 5000 steps. The 5000 images generated from each model maintained similar visual features as the input images. The generated images were resized to the original resolution (1920 × 1080 pixels) and cropped in the same manner as the training data, constructing a dataset with only the water surface portion.

#### 2.4.4. Evaluation Methods

This study employed AUROC (Area Under the Receiver Operating Characteristic Curve), F1 score, and anomaly heatmaps as evaluation metrics for model performance. AUROC is an indicator of how accurately the model distinguishes between normal and anomalous images, providing an overall evaluation of anomaly detection performance. AUROC is defined as the area under the Receiver Operating Characteristic (ROC) curve, which represents the relationship between the True Positive Rate (TPR) and the False Positive Rate (FPR) at various thresholds. The TPR indicates the proportion of actual positive data that is correctly predicted as positive by the model, while the FPR indicates the proportion of actual negative data that is incorrectly predicted as positive by the model.

The F1 score is the harmonic mean of precision and recall, which evaluates the model performance, particularly in imbalanced datasets. The F1 score is calculated using the following formula:

(1) $$F1Score\;=\;\frac{2\times Precision\times Recall}{\mathrm{Pr}ecision+Recall},$$

(2) $$Precision=\frac{TP}{TP+FP},$$

(3) $$Recall=\frac{TP}{TP+FN},$$

Precision indicates the sample proportion predicted as positive by the model that is actually positive. Recall indicates the proportion of actual positive samples that are correctly predicted as positive by the model. In the formula, TP (True Positive) refers to the samples correctly predicted as positive by the model, FP (False Positive) refers to the samples that are actually negative but incorrectly predicted as positive, and FN (False Negative) refers to the samples that are actually positive but incorrectly predicted as negative.

These evaluation metrics were combined to comprehensively assess the classification performance based on the ROC curve and the model’s performance on imbalanced datasets. Furthermore, anomaly heatmaps were generated for the visual evaluation of anomaly predictions using PaDiM. Anomaly heatmaps visualize the anomaly scores at each pixel of the input image using color, with regions of higher scores depicted in warmer colors. The anomaly score is a numerical indicator of how much each pixel deviates from the normal data distribution. This heatmap enables qualitative evaluation of the anomaly detection performance of PaDiM by checking whether the areas with concentrated anomaly scores align with the actual anomalous regions.

#### 2.4.5. Experimental Setup

The experiments were conducted using the Ubuntu operating system, utilizing an RTX 3090 GPU. The development environment consisted of Python 3.1, CUDA version 11.8, and PyTorch version 2.1.2. All libraries and tools are listed in Table 2.

## 3. Results

To improve the fish detection model performance, images rotated using AT, images processed with the NF in Adobe Photoshop, and normal images generated using SinGAN based on the five original normal images were added for training. Additionally, a new model was constructed using the normal images generated by SinGAN (SinGAN_pass) as training data, and a comparison of the model performance of the best models from each approach was conducted. Table 3 shows the total number of normal and anomalous images in the dataset used for training each model.

Table 4 presents the best evaluation metrics (champion data) extracted from the data obtained under different conditions for each method. The AUROC for Org was 0.836, while Org+AT increased to 0.942, and Org+NF achieved 0.940, indicating improved performance. However, Org+SinGAN showed the same AUROC as Org, and Org+SinGAN_pass resulted in a lower AUROC of 0.763. The model with the most notable improvement in AUROC was Org+AT, with an increase of 0.106.

The F1 Score for Org was 0.483, while Org+AT increased to 0.766 and Org+NF achieved 0.879, indicating a performance improvement. However, Org+SinGAN showed the same F1 Score as Org, while Org+SinGAN_pass resulted in a lower F1 Score of 0.474. The model with the most notable improvement in AUROC was Org+NF, with an increase of 0.396.

**Figure 2:**
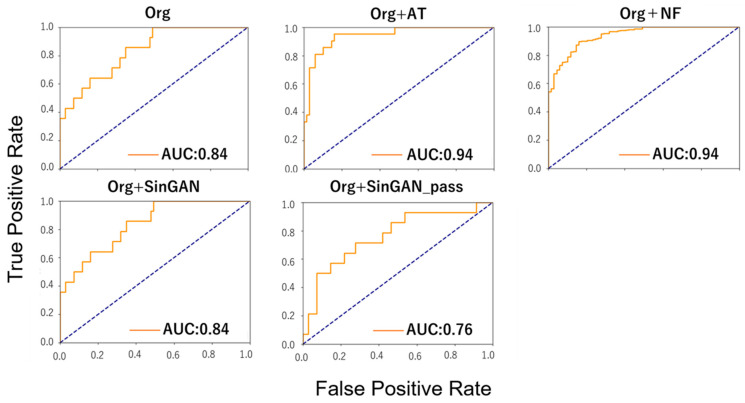
ROC curves for each model.

Figure 2 shows the ROC curves of the champion data for each method, which yielded the best evaluation metrics as presented in Table 4. The comparison of ROC curves across models showed that Org+NF achieved the highest TPR of approximately 0.55 at an FPR of approximately 0.0, indicating a strong early detection performance. Org+AT demonstrated a high performance, reaching a TPR of approximately 0.85 at an FPR of approximately 0.1. Org+SinGAN showed a gradual increase, achieving a TPR of approximately 0.84 at an FPR of approximately 0.4. The Org model exhibited a moderate curve, reaching a TPR of approximately 0.8 at an FPR of approximately 0.35. However, Org+SinGAN_pass showed a slower overall TPR increase, with a TPR of approximately 0.5 at an FPR of approximately 0.1, suggesting relatively lower early-stage detection compared to the other models.

**Figure 3:**
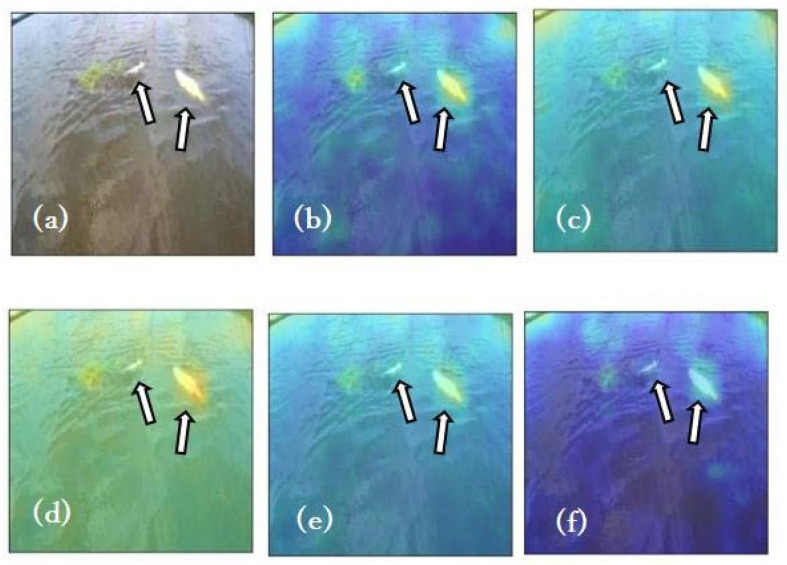
Visualization of anomaly prediction results. The figure presents (**a**) the original image used for inference, along with anomaly prediction heatmaps based on (**b**) Org, (**c**) Org+AT, (**d**) Org+NF, (**e**) Org+SinGAN, and (**f**) Org+SinGAN_pass. The arrows in the figure indicate fish.

Inference was performed on each of the constructed models using the test dataset, and the resulting anomaly heatmaps are presented in Figure 3. Compared to the anomaly heatmap of Org, Org+AT and Org+NF exhibited regions with higher anomaly scores at the fish locations, while relatively high anomaly scores were observed in the non-fish water surface areas (Figure 3c,d). For Org+SinGAN, the colors indicating the anomaly scores at the fish locations were not as prominent, and relatively high anomaly scores were observed in the water surface areas, excluding the fish (Figure 3e). Furthermore, Org+SinGAN_pass showed that the anomaly scores were suppressed at the fish locations and the water surface areas, excluding the fish (Figure 3f).

## 4. Discussion

### 4.1. Contributions and Limitations of This Study

This study aimed to improve fish detection accuracy (AUROC and F1 score) by incorporating additional image processing and generated images into the original dataset. The results confirmed that data augmentation using NF and AT-based rotation significantly enhanced performance, achieving an AUROC of 0.940 and an F1 score of 0.766, which compared favorably with related works reporting AUROC values from approximately 0.83 to 0.96 and F1 scores from 0.70 to 0.88 [14,21,30,33].

Evaluation of synthetic images generated by SinGAN and SinGAN_pass revealed that these images produced AUROC and F1 scores comparable to or lower than Org. Particularly, the SinGAN_pass-generated data, created under the limited condition of five original images, exhibited insufficient diversity, hindering further performance gains. Although the AUROC of SinGAN_pass was lower than Org, it demonstrated a certain performance level, given the constraints of data scale and quality.

An analysis of the anomaly score distribution showed that the Org+NF and Org+AT models generated anomaly scores at fish positions. However, an expansion of anomaly scores in non-fish water surface areas was observed, suggesting that data augmentation may have led to the unintended learning of non-fish features as anomalies.

Future works should focus on integrating advanced generative techniques, such as super-resolution and Stable Diffusion, to improve image quality and model generalization [32,34,35]. Moreover, accurate species classification requires deep learning models that can extract complex features from fish images. Convolutional Neural Networks (CNNs) have proven effective in capturing species-specific visual patterns by extracting subtle morphological details and color variations, which can enhance fish identification accuracy [36]. Additionally, detecting fish outside the camera’s field of view or beneath water surface remains challenging. Hence, systems that integrate multiple camera perspectives or leverage deep generative models, such as Generative Adversarial Networks (GANs), can supplement missing data by generating plausible representations of fish appearances and movements, facilitating wide-area monitoring [37].

### 4.2. Scope for Future Research

Data augmentation techniques, such as AT, NF, and SinGAN, hold considerable potential for enhancing fish distribution surveys using electrofishing boats and remotely operated vehicles (ROVs). These methods address image dataset limitations, particularly the scarcity of anomalous fish images, thereby improving the robustness and accuracy of detection models for rare or cryptic species. In the study area, *A. typus* and *M. nigricans* were designated as target species for conservation and eradication, respectively [27,28], underscoring the importance of reliable detection in electrofishing and ROV surveys. Moreover, these augmentation techniques facilitate real-time anomaly detection and automated monitoring systems, contributing to the conservation and sustainable management of aquatic ecosystems by providing detailed ecological information for improved fish management.

It is noteworthy that the evaluation dataset used in this study was limited by specific recording conditions. All video data were captured after 11:00 a.m. within a window of less than one hour, resulting in minimal variation in lighting conditions. However, realistic fish monitoring in lakes requires data collection at different times of day (e.g., early morning, midday, and evening) and under varied weather conditions (e.g., water surface glare, cloudy skies, and rainfall). In this study, we used time-of-day data augmentation to account for multiple daylight periods, and we consider that cloudy-sky conditions are covered by the morning and evening data used in our model. However, conditions such as water surface reflections and rainfall have not yet been evaluated. Incorporating data gathered under these additional conditions would likely improve the robustness and reliability of our detection models.

Furthermore, since this study focused solely on binary classification (i.e., the presence or absence of fish), the current system does not support species-level identification. Additionally, the inability to detect fish outside the camera’s field of view or beneath the water surface limits its applicability for large-scale ecological monitoring. Future improvements should extend the approach to include species classification using advanced deep learning models and integrate multi-angle and multi-sensor systems to expand the detection range and enhance overall monitoring capabilities.

## 5. Conclusions

This study demonstrates that integrating NF and AT-based rotation enhances fish detection performance in limited-data environments, achieving competitive AUROC and F1 scores. Although the use of SinGAN for synthetic image generation did not yield significant improvements, it underscored the need for further refinement of image generation techniques. Validated with fish video data from Lake Izunuma-Uchinuma, this study’s approach provides insights for environmental monitoring in complex aquatic settings.

Future work should focus on incorporating advanced generative methods to improve image quality and model generalization [32,34,35]. Moreover, the application of CNNs for species classification offers promising prospects for enhancing fish identification accuracy [36]. Additionally, addressing the challenge of detecting fish beyond the camera’s field of view through multi-angle and multi-sensor integration, potentially supported by GANs, will be critical for comprehensive ecological monitoring [37]. Together, these advancements can significantly contribute to the effective conservation and management of aquatic ecosystems.

## Figures and Tables

**Table 1:** Dataset details.

	Camera No.	Recording Time	Conversion Method	Normal Images	Anomalous Images	Resolution
Org	5	11:13~11:59	All frame conversion	3902	66	1390 × 550
Validation	5	11:13~11:59	All frame conversion	332	11	1390 × 550
Test	1	11:32~11:49	All frame conversion	0	100	1700 × 850
NFbase	5	11:21~11:46	1 frame per second	0	20	1920 × 1080
	5	10:00~10:06, 11:00~11:55	1 frame per minute	61	1	1920 × 1080

**Table 2:** Versions of libraries and tools used in the experiments.

Task	Library or Tool Name	Version
NF	Adobe Photoshop 2024	25.1.0
SinGAN	Github	https://github.com/kligvasser/SinGAN.git (accessed on 29 April 2025)
	Torch	2.5.0+cu124
	Torchvision	0.20.0+cu124
	Pillow	11.0.0
	tensorboardX	2.6.2.2
PaDiM	Anomalib	0.7.0
	Pydantic	2.4.2

**Table 3:** Total number of normal and anomalous images in the dataset.

Model Name	Normal Images	Anomalous Images	Resolution
Org	3902	66	1390 × 550
Org+AT	7804	102	1390 × 550
Org+NF	11,771	2130	1390 × 550
Org+SinGAN	13,902	66	1390 × 550
Org+SinGAN_pass	13,902	66	1390 × 550

**Table 4:** AUROC and F1 score for each model.

Model Name	AUROC	F1Score
Org	0.836	0.483
Org+AT	0.942	0.766
Org+NF	0.940	0.879
Org+SinGAN	0.836	0.483
Org+SinGAN_pass	0.763	0.474

## Data Availability

The data used in this study are not publicly available.

## Abbreviations

The following abbreviations are used in this manuscript:

YOLO	You Only Look Once
PaDiM	Patch Distribution Modeling
Org	Original dataset
AT	Affine transformation
NF	Neural Filter
SinGAN	Single Image Generative Adversarial Network
AUROC	Area Under the Receiver Operating Characteristic Curve
ROC	Receiver Operating Characteristic
TPR	True Positive Rate
FPR	False Positive Rate
TP	True Positive
FP	False Positive
FN	False Negative
CNNs	Convolutional Neural Networks
GANs	Generative Adversarial Networks

## References

[1] Naiman R., Latterell J. (2005). Principles for Linking Fish Habitat to Fisheries Management and Conservation. J. Fish Biol..

[2] Copp G.H. (1989). The Habitat Diversity and Fish Reproductive Function of Floodplain Ecosystems. Environ. Biol. Fish..

[3] Inoue M., Nakano S. (1994). Physical environment structure of a small stream with special reference to fish microhabitat. Jpn. J. Ecol..

[4] Hubert W.A., Murphy B.R., Willis D.W. (1996). Passive Capture Techniques. Fisheries Techniques.

[5] Moyle P.B., Vondracek B. (1985). Persistence and structure of the fish assemblage in a small California stream. Ecology.

[6] Fujimoto Y., Kurosaka K., Ojima D., Iwata M. (2008). Habitat Use and Shift of Two Sympatric Freshwater Sculpins (*Cottus pollux* and *Cottus hangiongensis*) during the Spawning and Non-spawning Seasons. J. Freshwat. Ecol..

[7] Fujimoto Y., Iwata M. (2005). Effect of Natural Light Conditions on the Use of Cover in Concrete Block Structures by Japanese Dace *Tribolodon hakonensis*. Fish. Sci..

[8] Holmes J.A., Cronkite G.M.W. (2008). Using Underwater Video to Count Migratory Salmon: A Cautionary Note. Fish. Res..

[9] Sward D., Monk J., Barrett N. (2019). A systematic review of remotely operated vehicle surveys for visually assessing fish assemblages. Front. Mar. Sci..

[10] Lyons J., Hateley J., Peirson G., Eley F., Manwaring S., Twine K. (2021). An assessment of hydroacoustic and electric fishing data to evaluate long term spatial and temporal fish population change in the River Thames, UK. Water.

[11] Mizuno K., Liu X., Asada A., Ashizawa J., Fujimoto Y., Shimada T. Application of a High-resolution Acoustic Video Camera to Fish Classification: An Experimental Study. Proceedings of the 2015 IEEE Underwater Technology (UT).

[12] Taberlet P., Coissac E., Hajibabaei M., Rieseberg L.H. (2012). Environmental DNA. Mol. Ecol..

[13] Oya Y., Kawasue K. (2010). Development of the Automatic Fish Monitoring Techniques Using Stereoscopic Images. Eco-Engineering.

[14] Hanson N., Ounsley J., Henry J., Terzić K., Caneco B. (2024). Automatic Detection of Fish Scale Circuli Using Deep Learning. Biol. Methods Protoc..

[15] Szegedy C., Toshev A., Erhan D. (2013). Deep Neural Networks for Object Detection. Adv. Neural Inf. Process. Syst..

[16] Zhao Z.Q., Zheng P., Xu S.T., Wu X. (2019). Object Detection with Deep Learning: A Review. IEEE Trans. Neural Netw. Learn. Syst..

[17] Wang N., Wang Y., Er M.J. (2020). Review on Deep Learning Techniques for Marine Object Recognition: Architectures and Algorithms. Control Eng. Pract..

[18] Sun M., Yang X., Xie Y. (2020). Deep Learning in Aquaculture: A Review. J. Comput..

[19] Redmon J., Farhadi A. (2018). YOLOv3: An Incremental Improvement. arXiv.

[20] Stavelin H., Rasheed A., San O., Hestnes A.J. (2021). Applying Object Detection to Marine Data and Exploring Explainability of a Fully Convolutional Neural Network Using Principal Component Analysis. Ecol. Inform..

[21] Berg P., Maia D., Pham M., Lefèvre S. (2022). Weakly Supervised Detection of Marine Animals in High Resolution Aerial Images. Remote Sens..

[22] Takahashi Y., Fujimoto Y. (2018). Survey of Shape and Area of Lake Izunuma-Uchinuma, Miyagi Prefecture, Japan Using Aerial Photographs Taken in 2007. Izunuma-Uchinuma Wetland Res..

[23] Fujimoto Y., Hayami H., Yokoyama J. (2019). Changes in Lakeside Vegetation Communities in Lake Izunuma-Uchinuma in Northeast Japan from 1976 to 2012. Wetland Res..

[24] Fujimoto Y., Shimada T., Inoue K., Takahashi Y., Hayami H. (2020). Below-average Water Level in Lake Izunuma-Uchinuma in Miyagi Prefecture, Japan, in the Winter of 2016/17 Induced Whooper Swan Feeding Activity, Leading to Reduced Coverage of Lotus Vegetation and Increased Dissolved Oxygen in the Water. J. Conserv. Ecol..

[25] Fujimoto Y., Kawagishi M., Shindo K. (2008). Freshwater Fishes in Lake Izunuma-Uchinuma Basin, Japan: Distribution Patterns of Native Species and Invasive Species. Izunuma-Uchinuma Wetland Res..

[26] Fujimoto Y., Shindo K. (2012). Small Scale Bitterling *Acheilognathus typus*: A Floodplain Fish Surviving in the Irrigation Pond. J. Ichthyol..

[27] Fujimoto Y., Takahashi K., Shindo K., Fujiwara T., Arita K., Saitoh K., Shimada T. (2021). Success in Population Control of the Invasive Largemouth Bass *Micropterus salmoides* through Removal at Spawning Sites in a Japanese Shallow Lake. Manag. Biol. Invas..

[28] Fujimoto Y., Takahashi K., Shinto K., Saito K., Mitsuka M., Shimada T. (2021). Recovery of the Endangered Bitterling *Acheilognathus typus* in Lake Izunuma-Uchinuma after the Removal of Largemouth Bass (*Micropterus salmoides*). J. Ichthyol..

[29] Fujimoto Y., Fujimoto Y., Shimada T., Takahashi K., Saitoh K. (2013). Comparison of the Efficacy of Electrofishing Boat, Setnet, and Gillnet for the Control of Largemouth Bass. Manual of Control of Alien Fish and Recovery of Native Fishes for the Restoration of Lake Ecosystems: Based on the Studies in Lake Izunuma-Uchinuma.

[30] Defard T., Setkov A., Loesch A., Audigier R. (2020). PaDiM: A Patch Distribution Modeling Framework for Anomaly Detection and Localization. arXiv.

[31] Krizhevsky A., Sutskever I., Hinton G.E. (2012). ImageNet Classification with Deep Convolutional Neural Networks. Adv. Neural Inf. Process. Syst..

[32] Shaham T.R., Dekel T., Michaeli T. (2019). SinGAN: Learning a Generative Model from a Single Natural Image. arXiv.

[33] Bergmann P., Löwe S., Fauser M., Sattlegger D., Steger C. (2019). Improving Unsupervised Defect Segmentation by Applying Structural Similarity to Autoencoders. Proceedings of the 14th International Joint Conference on Computer Vision, Imaging and Computer Graphics Theory and Applications (VISIGRAPP 2019).

[34] Shorten C., Khoshgoftaar T.M. (2019). Image Data Augmentation for Deep Learning: A Survey. J. Big Data.

[35] Rombach R., Blattmann A., Lorenz D., Esser P., Ommer B. High-Resolution Image Synthesis with Latent Diffusion Models. Proceedings of the IEEE/CVF Conference on Computer Vision and Pattern Recognition (CVPR) 2022.

[36] Zhang L. (2017). Deep Learning for Fish Classification: A Survey. J. Mar. Sci. Eng..

[37] Goodfellow I.J., Pouget-Abadie J., Mirza M., Xu B., Warde-Farley D., Ozair S., Courville A., Bengio Y. (2014). Generative Adversarial Networks. arXiv.

